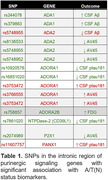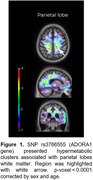# A/T(N) Status Biomarkers Associate with Polymorphisms in Purinergic Signaling Genes

**DOI:** 10.1002/alz.095194

**Published:** 2025-01-09

**Authors:** Luciana Rocha Costa, Marco Antônio de Bastiani, Luiza Santos Machado, Wyllians Vendramini Borelli, Andrea Benedet, Eduardo R. Zimmer, Diogo O. Souza, Débora Guerini de Souza

**Affiliations:** ^1^ Federal University of Rio Grande do Sul, Porto Alegre, Rio Grande do Sul Brazil; ^2^ University of Gothenburg, Gothenburg Sweden; ^3^ Memory Center, Hospital Moinhos de Vento, Porto Alegre, RS Brazil; ^4^ Federal University of Rio Grande do Sul (UFRGS), Porto Alegre, RS Brazil; ^5^ Brain Institute of Rio Grande Do Sul, PUCRS, Porto Alegre, RS Brazil

## Abstract

**Background:**

Purinergic signaling is vital in various cellular processes like neuroinflammation, synaptic transmission, Aβ clearance, and tau phosphorylation regulation. This study aims to examine if SNPs in purinergic signaling genes are associated to A/T(N) status biomarkers in Alzheimer’s Disease through Genome Wide Association Studies.

**Method:**

The SNPRelate package was used to analyze SNPs in genes related to purinergic signaling and A/T(N) markers. We included 152 cognitively unimpaired and 359 cognitively impaired individuals from the ADNI cohort. Generalized linear models were applied to investigate associations between independent variables (SNPs, APOE4 status, sex, age, education, and diagnosis), and A/T(N) markers, specifically tau status (CSF/Plasma ptau181), amyloid status (CSF Aβ and AV45‐PET ratio of cortical grey matter/whole cerebellum), and neurodegeneration status (average FDG‐PET metaROI). We also conducted a voxel‐wise linear regression testing the association between [18F]FDG metabolism and SNP carriership, corrected by sex and age. The analysis was corrected for multiple comparisons using the cluster‐wise random field theory method (significant t>3.11, p<0.001, df = 495).

**Result:**

We identified 13 SNPs in the intronic region of purinergic signaling genes that had a significant association with A/T(N) status biomarkers (Table 1). CSF Aβ levels were associated with polymorphisms in the adenosine deaminase 1 (ADA1) and 2 (ADA2) genes. AV45‐PET binding was associated with SNPs in ADA2, ADORA1, and P2×1 genes. CSF ptau181 levels were associated with polymorphisms in the ADORA1, NTPDase‐2/CD39L1, and pannexin 1 gene. Increased FDG uptake was associated with a polymorphism in the ADORA2B gene. Finally, a polymorphism in the ADORA1 gene presented a positive association with [18F]FDG metabolism in the parietal lobe white matter (tmax = 3.97, p<0.0001) (Figure 1).

**Conclusion:**

Multiple SNPs within genes related to purinergic signaling have been identified to be linked with A/T(N) status markers. These results underscore the significance of purinergic signaling in cellular processes relevant to brain homeostasis, suggesting potential implications for Alzheimer’s disease (AD). Consequently, these findings highlight promising cellular mechanisms underlying the development of dementia.